# Model based prediction of age-specific soil and dust ingestion rates for children

**DOI:** 10.1038/s41370-021-00406-5

**Published:** 2022-01-17

**Authors:** Haluk Özkaynak, Graham Glen, Jonathan Cohen, Heidi Hubbard, Kent Thomas, Linda Phillips, Nicolle Tulve

**Affiliations:** 1 ICF, Durham, NC 27713 USA; 2 U.S. EPA, Office of Research and Development, Research Triangle Park, NC 27711 USA; 3 U.S. EPA, Office of Research and Development, Retired, Washington, D.C., USA

**Keywords:** Children, Soil, Dust, Ingestion, SHEDS-Soil/Dust Model

## Abstract

**Background:**

Soil and dust ingestion can be a primary route of environmental exposures. Studies have shown that young children are more vulnerable to incidental soil and dust ingestion. However, available data to develop soil and dust ingestion rates for some child-specific age groups are either lacking or uncertain.

**Objective:**

Our objective was to use the Stochastic Human Exposure and Dose Simulation Soil and Dust (SHEDS-Soil/Dust) model to estimate distributions of soil and dust ingestion rates for ten age ranges from infancy to late adolescents (birth to 21 years).

**Methods:**

We developed approaches for modeling age groups previously not studied, including a new exposure scenario for infants to capture exposures to indoor dust via pacifier use and accounting for use of blankets that act as a barrier to soil and dust exposure.

**Results:**

Overall mean soil and dust ingestion rates ranged from ~35 mg/day (infants, 0–<6 m) to ~60 mg/day (toddlers and young children, 6m–<11 yr) and were considerably lower (about 20 mg/day) for teenagers and late adolescents (16–<21 y). The pacifier use scenario contributed about 20 mg/day to the median dust ingestion rate for young infants. Except for the infant age groups, seasonal analysis showed that the modeled estimates of average summer mean daily total soil plus dust ingestion rates were about 50% higher than the values predicted for the winter months. Pacifier use factors and carpet dust loading values were drivers of exposure for infants and younger children. For older children, influential variables included carpet dust loading, soil adherence, and factors that capture the frequency and intensity of hand-to-mouth behaviors.

**Significance:**

These results provide modeled estimates of children’s soil and dust ingestion rates for use in decision making using real-world exposure considerations.

**Impact statement:**

The parameterization of scenarios to capture infant soil and dust ingestion and the application of SHEDS-Soil/Dust to a broader age range of children provides additional estimates of soil and dust ingestion rates that are useful in refining population-based risk assessments. These data illuminate drivers of exposure that are useful to both risk management applications and for designing future studies that improve upon existing tracer methodologies.

## Introduction

Incidental ingestion of chemical and biological agents found in soil and dust poses health risks to children. As a result, accurate quantification of incidental ingestion rates is critical for assessing both contaminant intake and associated human health risks from soil and dust contact. The U.S. EPA’s Exposure Factors Handbook (EFH) [1, 2] reviews and summarizes available studies for estimating daily soil and dust ingestion rates for children.

Three primary methodologies are used to estimate soil and dust ingestion rates for children: trace-element based mass balance approach, time-activity pattern modeling, and to a lesser extent biokinetic modeling approaches [3]. Each methodology has its respective uncertainties.

Most publications referenced in the EFH are based on studies of young children (3–<6 years old) using the trace element mass balance estimation approach with aluminum and silicon being the most widely used and considered among the more reliable tracers. Children’s average soil and dust ingestion rates typically range from 10 to over 100 mg/day [2, 4–11]. As described in Doyle et al. [12], Moya and Phillips [3], and Özkaynak et al. [13], the tracer element mass balance estimation approach has several limitations, including uncertainty in accounting for the contribution of dietary ingestion of tracer elements, intra- and inter-subject variation in gastro-intestinal transit times, missing observations, negative ingestion values produced from applying a mass balance model, recovery and stability issues with some tracers, errors due to not accounting for other sources of tracer intake, and most importantly, the inability to differentiate between soil and dust intake. This is an important differentiation because soil and dust are likely to contain different contaminant profiles. Indoor dust is also likely to be partially comprised of tracked in soil; understanding both soil and dust ingestion rates are critical to quantify total contaminant intake via this pathway. Additionally, study findings are not readily comparable with each other due to differences in the age groups selected. Furthermore, they do not provide soil and dust ingestion rates for all pertinent child-specific age groups, as specified in the EFH [1, 2].

The relatively few time-activity pattern [13–15] and biokinetic-based [16] modeling methodologies provide mechanistically-based soil and dust ingestion rates for children. However, model formulation and measurement-based input and parameter uncertainties could lead to prediction errors based on the use of these approaches. Methods to characterize the nature and magnitude of different types of particle or chemical ingestion related uncertainties have been discussed in Xue et al. [17], Zartarian et al. [18], Özkaynak et al. [13], Hsi et al. [19] and Li et al. [20].

As an integral part of the SHEDS-Multimedia model, the SHEDS-Soil/Dust model, which uses the time activity method, has been used reliably to estimate young children’s (3–<6 years old) soil and dust ingestion rates to arsenic from chromated copper arsenate (CCA) treated decks and playsets, indoor applications of chlorpyrifos and permethrin, and numerous semi-volatile chemicals found in consumer products [13, 18, 21–24]. In particular, the model evaluation studies have been shown to be successful based on comparison of predicted parent or metabolite chemical concentrations in blood or urine to available measurement data.

The main goal of this research was to broaden the soil and dust ingestion predictions for a greater number of age groups from birth to 21 years (specifically, the age ranges of 0 to <1 m, 1 to <3 m, 3 to <6 m, 6 m to <1 y, 1 to <2 y, 2 to <3 y, 3 to <6 y, 6 to <11 y, 11 to <16 y, 16 to <21 y), using the SHEDS-Soil/Dust model while refining the model code to include an algorithm that incorporates a blanket modification and pacifier exposure scenario to estimate soil and dust ingestion rates for infants.

## Methods

### Overview of SHEDS-Soil/Dust Model

We started with the SHEDS-Soil/Dust model code published in Özkaynak et al. [13] with updates in the Residential module [25] of the SHEDS-Multimedia Multipathway model (Version 4) code (https://www.epa.gov/sites/production/files/2015-02/documents/shedsresidential_techmanual_2012.pdf).

**Fig. 1:**
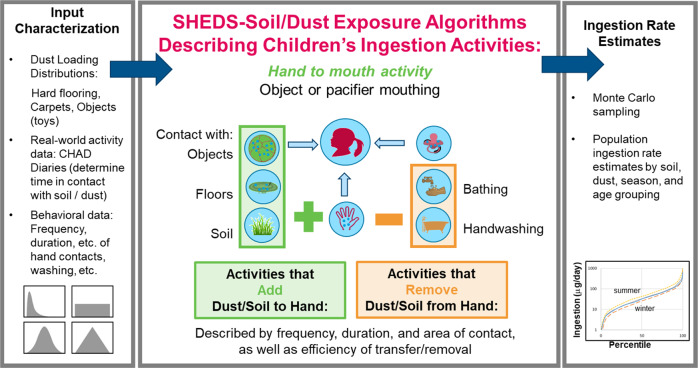
Components of the SHEDS-Soil/Dust ingestion model. Data inputs and exposure algorithms are used to describe activities that add and remove dust and soil from the hands resulting in ingestion estimates. The panels are described in more detail in the text.

Figure 1 shows the main components of the SHEDS-Soil/Dust model. Further details on the model can be found in the Supplemental Information (SI) and Özkaynak et al. [13]. To begin, the SHEDS-Soil/Dust model first characterizes indoor dust loadings on hard floors, soft floors (i.e., carpet), and objects (e.g., toys) by sampling the specified distributions for these variables. The model relies on activities from the latest version of the CHAD diaries (https://www.epa.gov/healthresearch/consolidated-human-activity-database-chad-use-human-exposure-and-health-studies-and) to determine the time spent in contact with either dust (indoors) or soil (outdoors) (left-hand box of Fig. 1). Data inputs are used to model a child’s activity pattern that puts them in contact with dust and soil, either through hand contact followed by hand-to-mouth contact or direct contact of objects with the mouth (e.g., mouthing of toys that have contacted floor or carpet dust). Dust and soil removal from the hand surface via bathing, handwashing, and hand-to-mouth contact are also modeled (central box in Fig. 1). This provides the SHEDS-Soil/Dust model with the ability to generate separate intake estimates for indoor dust and outdoor soil, along with combined estimates.

The SHEDS-Soil/Dust model is a stochastic and population-based model, meaning it relies on distributions of inputs rather than point values, and the model simulates a representative population in each age group (in this exercise, there were 5000 simulated children per age bin; shown in the right-hand box of Fig. 1). All age groups were run for one year per simulated person using the 8-diary approach (i.e., by sampling diaries from available weekday and weekend days for each of the four seasons, where winter is defined as December–February, spring is March–May, summer is June–August, and fall is September–November) to predict annual average daily soil and dust ingestion rates. In addition, we conducted separate season-specific model runs to examine temporal variability in model predictions over the course of a year. The SHEDS-Soil/Dust model assigns an age to each simulated person and assumes it does not change during the simulation. This is reasonable for children aged one year and above, but infants below one year would not remain in the same age group for a full year. For this reason, we performed separate runs of the model for each of four age groups below one year (i.e., 0–<1 m, 1–<3 m, 3–<6 m, and 6–<12 m) for a full year to estimate annual averages. Running the SHEDS-Soil/Dust model for a full year allows for both annual and seasonal statistics to be calculated.

### Input variable distributions for infants under 1 year old

To model infants’ soil and dust ingestion rates, new exposure algorithms for infants under one-year old were required. We assumed the main exposure pathway for these very young age children results from putting dusty pacifiers into the mouth. We also assumed that pacifiers fall onto a variety of items, including carpet, hard floors, blankets, or clothing, picking up a portion of the dust from these surfaces after each fall. Subsequent insertions of the pacifier into the mouth would then lead to incidental dust ingestion. Modeling this scenario required information on how much blankets affect dust transfer to the pacifier, number of pacifier drops, pacifier loading rates, and dust transfer into the mouth during sucking activities.

#### Blankets

The “blanket” modification reflects the fact that young children may be placed in a crib, playpen, or stroller, or on a blanket, cloth, or sheet for periods of time throughout the day. In our analysis, we collectively refer to all these barriers as “blankets”.

We derived two new input variables for SHEDS-Soil/Dust model to account for the blanket modification (Table 1a). F_blanket is the protective barrier factor provided by the blanket, when used. The other input is P_blanket, the probability of a blanket being used at a given time. Literature-based values were not identified for these variables. Based on personal experience, we assigned the F_blanket variable a mean value of 0.25, with an associated uniform distribution ranging from 0.1 to 0.4. Coincidentally, this 4-fold dust reduction factor is consistent with the findings from Roberts et al. [26] and Yu et al. [27], in terms of reduction of carpet dust loadings after cleaning. It is likely that scenarios exist in which a blanket is in fact dustier or dirtier (in the case of soil) than the floor. We believe this is more likely to be the case for older children who carry a blanket around. In this scenario, mouthing of the blanket would fall under “object mouthing” described in the Object to Mouth Area and Object to Mouth Frequency subsections. Based on our collective parenting experience, P_blanket is assumed to be quite high below 6 months and zero above two years.

**Table 1:** Input variable probability distributions required to model pacifier exposure: (a) distributions used for blanket variables, (b) non-age dependent distributions used for pacifier variables, and (c) age-dependent distributions used for pacifier variables.

(a)			(b)		(c)		
Variable	Age groups	Distribution*	Variable	Distribution*	Variable	Age group	Distribution*
P_blanket (probability of blanket use)	0–<1 m, 1–<3 m, 3–<6 m	Uniform (0.8, 1.0)	Pacifier_size (cm^2^, size of pacifier)	Uniform (8, 11)^1^	Pacifier_drop (hr^-1^, frequency of pacifier dropping)	0–<1 m, 1–<3 m, 3–<6 m	Normal (8, 3)^5^
	6–<12 m	Uniform (0, 1)	Pacifier_frac_hard (fraction of pacifier drops onto hard surface)	Point (0.25)^2^		6–<12 m	Normal (5, 2)^6^
	1–<2 y	Uniform (0, 0.2)	Pacifier_frac_soft (fraction of pacifier drops onto soft surface)	Uniform (0.25, 0.5)^2^		1–<2 y	Normal (2, 1)^5^
	2 y or older	Point (0)	Pacifier_transfer (fraction of dust transferred from floor to pacifier)	Uniform (0.25, 0.75)^3^	P_Pacifier (probability of pacifier use)	0–<1 m, 1–<3 m, 3–<6 m	Uniform (0.8, 1)
F_blanket (protective barrier factor)	All	Uniform (0.1, 0.4)	Pacifier_washing (efficiency of pacifier washing)	Point (0)^4^		6–<12 m	Uniform (0.5, 1)

*Parentheticals indicate the parameters for the statistical distribution used for the given variable. The parameters, by distribution, are: normal–mean, SD; uniform–min, max; point–point value.

^1^Based on measurement of commercially available pacifiers.

^2^Professional judgment.

^3^Extrapolated from published data.

^4^Conservative assumption.

^5^Extrapolated from data presented in Tsou et al. [31].

^6^Presented in Tsou et al. [31].

The blanket modification acts to modify the other soil/dust exposure scenarios by reducing the amount of soil or dust to which a child may be exposed when the blanket is present. In implementation, P_blanket is given a new probability check on each diary event, but F_blanket is set once per person.

#### Approach used to model exposures due to pacifier use

We devised a new exposure scenario for very young children related to pacifier use, which is distinct from other object-to-mouth contacts. This resulted in seven new input variables to the SHEDS-Soil/Dust model. These variables, listed in Tables 1b, c, describe pacifier size, the fractional areas of a pacifier that encounter hard and soft surfaces when dropped, and the amount of dust transferred to the pacifier when in contact with a surface. These variables are described in more detail in the SI.

Pacifier_size (cm^2^): based on actual measurements of commercially available pacifiers. Distribution was set to uniform between 8 and 11 cm^2^.

Pacifier_frac_hard and Pacifier_frac_soft: are the fractional areas (of the pacifier nipple) that contact the floor when the pacifier is dropped on hard or soft surfaces, respectively. We conducted an experiment (using a pliable plastic food cutting board lightly dusted with flour) to determine the potential surface area of the pacifier which may come in contact with either a hard or soft surface when dropped. Based on our results, we selected a point value of 0.25 when the pacifier falls on a hard surface and a uniform distribution from 0.25 to 0.50 when the pacifier falls on a soft surface.

Pacifier_transfer reflects the dust fraction transferred from the floor to the pacifier. Pacifiers are expected to only be used indoors or outdoors on a barrier, such as a blanket. For this reason, soil to pacifier transfer is not considered. This is recognized as a conservative assumption with regards to soil ingestion. We were unable to identify any data in the literature for dust transfer efficiencies to pacifiers. However, there were data on transfer efficiencies of pesticides, fluorescein-tagged dust and fluorescent tracers (surrogates used for different pesticides) onto dry and wet hands or fingers. Since pacifiers are made of smooth silicone or rubber, they offer higher contact area to dust particles than contoured hands or fingers. Moreover, pacifier tips are wet by their use. These features enhance the dust transfer rates onto dropped pacifiers. Thus, we chose a similar upper value of results presented in Rodes et al. [28], Beamer et al. [29] and Cohen-Hubal et al. [30] for wet hands/fingers as the pacifier_transfer variable, representing a single contact. However, when pacifiers are dropped, they roll over on clothing, furniture or floor, and different parts of the pacifier tip make multiple contacts with the surfaces touched. As shown in Rodes et al. [28] multiple contacts with surfaces carrying dust can significantly increase dust transfer to hands/fingers. With these considerations in mind, we chose an upper value of 0.75 for the pacifier_transfer variable. We then specified the pacifier_transfer variable to be a uniform distribution between 0.25 and 0.75.

Pacifier_washing: a composite of the probability of cleaning the pacifier after it falls and the efficiency of cleaning. Setting this variable equal to zero means washing never occurs. However, we anticipate that most foreign matter may be removed even with minor rinsing. Pacifiers fall many times per day and not everyone is careful about cleaning it after each drop. For our modeling analysis, we set the Pacifier_washing variable equal to zero, with the understanding that the resulting estimates are conservative.

#### Exposure equations used for the pacifier scenario

Because of the pacifier exposure scenario, the following new equations were implemented in the SHEDS-Soil/Dust code.

Amount of dust transferred per drop is:

$${{{{{{{\mathbf{Dust}}}}}}}}\_{{{{{{{\mathbf{per}}}}}}}}\_{{{{{{{\mathbf{drop}}}}}}}} =	\; {{{{{{{\mathbf{surface}}}}}}}}\_{{{{{{{\mathbf{dust}}}}}}}}\_{{{{{{{\mathbf{loading}}}}}}}} \times {{{{{{{\mathbf{Pacifier}}}}}}}}\_{{{{{{{\mathbf{size}}}}}}}} \times {{{{{{{\mathbf{Pacifier}}}}}}}}\_{{{{{{{\mathbf{frac}}}}}}}} \\ 	\times {{{{{{{\mathbf{Pacifier}}}}}}}}\_{{{{{{{\mathbf{transfer}}}}}}}}\\ (\mu g/drop) =	 \; (\mu g/cm^2) \times \left( {cm^2} \right)\times \left( - \right)\quad \times \left( {fraction/drop} \right)$$

Surface_dust_loading is modified by the blanket, if present. Pacifier_frac is set to either pacifier_frac_hard or pacifier_frac_soft depending on floor type.

Amount of dust ingested on this diary event is:

$${{{{{{{\mathbf{Exp}}}}}}}}\_{{{{{{{\mathbf{pacifier}}}}}}}}\_{{{{{{{\mathbf{event}}}}}}}} =	\; {{{{{{{\mathbf{Dust}}}}}}}}\_{{{{{{{\mathbf{per}}}}}}}}\_{{{{{{{\mathbf{drop}}}}}}}} \times {{{{{{{\mathbf{Pacifier}}}}}}}}\_{{{{{{{\mathbf{drop}}}}}}}} \times {{{{{{{\mathbf{event}}}}}}}}\;{{{{{{{\mathbf{duration}}}}}}}} \\ 	\times \left( {{{{{{{{\mathbf{1}}}}}}}} - {{{{{{{\mathbf{Pacifier}}}}}}}}\_{{{{{{{\mathbf{washing}}}}}}}}} \right)\\ \left( {\mu g/diary\;event} \right) =	\; \left( {\mu g/drop} \right)\ \times \left( {drops/hr} \right)\; \times \left( {hr/diary{{{{{{{\mathrm{ }}}}}}}}event} \right) \times \left( - \right)$$

The event duration is sampled from the CHAD time-activity database and can range from 1 minute (e.g., handwashing) to one hour (e.g., playing or sleeping, which is reported in one hour increments). Daily dust ingestion from pacifier use is the sum of Exp_pacifier_event over the relevant diary events (those when the child is indoors and awake). A unit conversion is required because surface dust loadings measure mass in micrograms, but daily ingestion is in milligrams per day. Thus,

$${{{{{{{\mathbf{Exp}}}}}}}}\_{{{{{{{\mathbf{pacifier}}}}}}}}\_{{{{{{{\mathbf{daily}}}}}}}} 	= \; {\sum} {\left( {{{{{{{{\mathbf{Exp}}}}}}}}\_{{{{{{{\mathbf{pacifer}}}}}}}}\_{{{{{{{\mathbf{event}}}}}}}}} \right)/{{{{{{{\mathbf{1000}}}}}}}}} \\ \left( {mg/day} \right) 	= \; \left( {\mu g/day} \right)/\left( {\mu g/mg} \right)$$

Children below one year of age are assumed to have no soil contact. If they are outdoors, they are usually being carried or pushed in a stroller and cannot reach the ground. In the 1 y (i.e., 1 to <2 yr old) age group, there is still occasional pacifier use, and these children have reduced access to the ground (see the later discussion on soil adherence inputs). In the rare event a young child (1–<2 yr) may be playing outdoors, a soil contact equation is used. In this case, the soil adherence factor (in μg/cm^2^) replaces the product of the variables surface_dust_loading and pacifier_transfer (this product is also in μg/cm^2^ and measures the amount of dust adhering to each affected square centimeter of the pacifier each time it is dropped).

#### Frequency of pacifier use

We used the data published by Tsou et al. [31] and from its Supplemental Information to estimate the frequency of young children’s mouth contacts with a pacifier. Mean pacifier contact frequency for all 66 children was 0.82 contacts per hour. Recalculating the mean pacifier contact frequency for the 13 children reported to always use pacifiers gives a value of ~0.82 × 66/13, i.e., roughly 5 contacts per hour. Because we assumed these children always use pacifiers, the frequency of use (i.e., new insertion into mouth) is also equal to frequency of drop rates, i.e., Pacifier_drop.

We also assumed the pacifier drop rate was greatest between 0 and <6 months, least between 1 and <2 years, and average of 5 drops per hour for 6–<12 months. Table 1c lists the pacifier contact/drop frequencies that we assumed during waking hours by age group.

#### Probability of pacifier use

Table 1c lists the distributions for P_Pacifier. P_Pacifier is the probability that a young child is using a pacifier during a given diary event. We used the information presented in the SI on mouthing behaviors by development age to inform our development of this variable, and the data presented in Tsou et al. [31] data to estimate this variable. We assumed pacifier use was highest for children below 6 months of age and then decreases as children age. We also assumed no pacifier use once a child reaches 2 years of age (even though there may be exceptions to this generalization).

### General model assumptions and distributions for input variables

To run the SHEDS-Soil/Dust model, the user must specify a simulation start date, length, population size, and ages to be simulated. Ten different child-specific age groups were modeled to estimate soil and dust ingestion rates. For our analysis, we selected a population of 5000 persons for each age group to ensure model stability. We used the same distribution for surface area of hands used in previous SHEDS-Soil/Dust model runs [13]. We assumed a 50% chance that an individual may contact a bare floor versus a carpeted floor when they contact an indoor floor surface. We also assumed dust to be present in all indoor locations and soil to be present in all outdoor locations. All diary events (except for sleeping and bathing events) had the possibility of hand contact with either dust or soil.

#### Age-independent model variables

Table S1 summarizes the selected age-independent values for the model input variables relevant for estimating soil and dust ingestion rates for children of all age groups. Sources of the variable-specific data and the rationale used in developing these distributions can be found in Özkaynak et al. [13].

#### Age-dependent model input variables

Variables that describe mouthing behavior (hand- and object-to-mouth intensity, described by the frequency and area of contacts) plus the adherence of soil to the hand are assumed to be dependent on age and driven by the stages of pediatric development. Information used to inform the choices of variables are presented in the SI. Data used to develop the variables are presented in the following five subsections.

##### Soil-adherence

Adherence_soil represents a soil-skin adherence factor and reflects the accumulated mass of soil that is transferred onto skin, expressed as a loading. We assumed very young children did not have direct contact with soil and therefore set the soil adherence to zero for age groups below one year and assumed the soil adherence distribution for the 1 y age group was a lognormal distribution with a geometric mean of 0.055 mg/cm^2^ and a geometric standard deviation of 2.0. For all children in the 2 y age group and beyond, the soil adherence was assumed to be lognormal with a geometric mean of 0.11 mg/cm^2^ and a geometric standard deviation of 2.0 per Özkaynak et al. [13]. Table S2 summarizes the soil adherence distributions used.

##### Hand-mouth fraction

Hand_mouth_fraction is the fraction of hand area of one hand contacting the inside of the mouth allowing soil and dust to be removed. A rough guideline is that the palm is 25%, the back of the hand 25%, and each finger is 10% [32]. We set this variable to 10% to represent when a child is thumb sucking as in Özkaynak et al. [13].

For this variable, we used either the data in Özkaynak et al. [13] or fitted age-specific beta distributions to the Tsou et al. [33] results. Since published information was not available for either the very young or older aged children, we extrapolated available values to estimate this data input. Table S3 provides the age-specific distributions for the fraction of hand area mouthed, along with our assumptions.

##### Hand-mouth frequency

Hand_mouth_freq is the average frequency of hand-mouth contacts per hour while awake (Table S4). Data for age groups 3 m to <3 y were taken from Xue et al. [21]. Values for the remaining age categories were estimated by extrapolating from the nearest age category (using the mean values of the distributions as the bases) for which we had quantitative information using Xue et al. [21] and Xue et al. [34]. For each extrapolated value, we considered children’s ages, developmental stage, and pediatric guidance in making our estimates. For example, children’s hand-to-mouth and object-to-mouth behaviors increase sharply and steadily up to 2 years of age. After age two, these contact rates start to decrease gradually till about 6 years of age, after which they start to drop off very rapidly.

##### Object to mouth area

Object_mouth_area is the area of an object inserted into the mouth (Table S5). In previous SHEDS-Soil/Dust runs, this variable was set to an exponential distribution (minimum = 1, mean = 10, and maximum = 50 cm^2^) [13]. Values were extrapolated for children younger than 1 to 3 years old using the calculated relative differences in hand areas for children in the 3–<6 y age group [35]. We used hand size ratios to adjust the mean and maximum values. We also assumed that older children typically do not engage in object mouthing activities [13].

##### Object to mouth frequency

Object_mouth_freq is the frequency at which objects are moved into the mouth (Table S6). This variable was fit to available data on children’s object-to-mouth frequency data from Xue et al. [34] separately for indoor and outdoor locations. We assumed the youngest age group of children do not pick up objects lying on the floor or ground (object_mouth_freq = zero).

Using the data provided in Tsou et al. [31, 33] on object_mouth contacts with pacifier and separately with total non-dietary contacts, we adjusted the object_mouth contact exposure predictions in Özkaynak et al. [13] by an average of 4% to account for the pacifier portion of the total non-dietary object contacts based on the information presented on the frequency and probability of pacifier use.

#### Model runs and processing of outputs

The model was run separately for each age group (sample size = 5000 individuals for a time period of one full year). Males and females were run together due to the lack of sex-dependent input distributions. The code automatically provides annual (one record per person) and seasonal (four records per person) summaries for each run. Each record provides demographic (age, sex, weight, height, and skin surface area) information and several exposure metrics (hand-to-mouth ingestion, object-to-mouth ingestion, and pacifier-mediated ingestion, with separate estimates for soil and dust and a total for soil and dust) averaged over the specific time period (either season or year). We report six population statistics (mean, standard deviation, geometric mean, geometric standard deviation, median, and 95th percentile) for dust, soil, and total (dust + soil) ingestion rates for both seasonal and annual averages for each age group.

#### Sensitivity analysis

We used the approach described in Özkaynak et al. [13] to assess the sensitivity of predicted modeling results to choices made in the specification of input distributions to the SHEDS-Soil/Dust model. This method evaluates which model inputs are key drivers of the model results and contribute to greatest variability and/or uncertainty to our predicted results.

Furthermore, we performed an additional sensitivity analysis on three uncertain parameters in the new pacifier scenario: pacifier_washing, pacifier_drop, and pacifier_transfer to assess the impact of the chosen distributions. The specifics of the sensitivity analysis performed are discussed in the SI.

## Results

Basic summary statistics for the predicted annual mean daily soil, dust, and total soil plus dust ingestion rates for each age group are presented in Table 2. Briefly, the model predictions for annual mean (median) daily total soil plus dust ingestion rates for newborns and infants 0 to <6 months old were about 35 (20) mg/day. Because children this young often do not encounter outdoor soil, their predicted incidental ingestion rates are only attributed to indoor dust. Slightly higher annual (again largely from indoor dust) total soil plus dust ingestion rates of ~45 (30) mg/day were predicted for children aged 6 months to <2 years. Annual mean (median) daily total soil plus dust ingestion rates for children aged 2 to <11 years old were estimated to be around 55 (35) mg/day. For children older than 11 years, the predicted total soil plus dust ingestion rates declined, most likely due to decreased hand-to-mouth and object-to-mouth activities. The modeled mean (median) annual total soil plus dust ingestion rates for ages 11 to <16 years were 44 (21) mg/day and for ages 16 to <21 years were 23 (8) mg/day.

**Table 2:** Annual mean daily total dust and soil ingestion rate (mg/day) predictions.

Age group	Dust plus soil ingestion						Dust ingestion						Soil ingestion					
	Mean	Std. Dev	GM	GSD	Median	95th %ile	Mean	Std. Dev	GM	GSD	Median	95th %ile	Mean	Std. Dev	GM	GSD	Median	95th %ile
0–<1 m	32	44	19	2.9	18.9	103	32	44	19	2.9	18.9	103	0	0	.	.	0	0
1–<3 m	36	53	21	2.8	20.4	116	36	53	21	2.8	20.4	116	0	0	.	.	0	0
3–<6 m	37	47	23	2.7	22.6	112	37	47	23	2.7	22.6	112	0	0	.	.	0	0
6 m–<1 y	44	70	26	2.8	25.8	133	44	70	26	2.8	25.8	133	0	0	.	.	0	0
1–<2 y	48	57	32	2.4	31.4	140	37	54	23	2.6	22.5	119	10	16	4.8	3.8	5.1	38
2–<3 y	52	65	32	2.9	33.9	158	26	45	14	3.1	14.4	83	27	39	12	4.2	13.7	98
3–<6 y	59	70	36	2.9	36.9	190	28	40	15	3.2	15.8	94	31	48	14	4	14.8	118
6–<11 y	56	75	30	3.3	32.5	187	25	38	13	3.4	13.4	87	31	54	13	4.3	14.2	114
11–<16 y	44	71	20	3.9	21.4	161	21	41	8.8	4.1	9.6	78	23	45	7.6	5.2	8.3	95
16–<21 y	23	49	7.3	5.1	7.6	99	11	27	3.5	5.3	3.8	46	12	33	2.3	7.6	2.6	51

For most age groups, the median and geometric mean as well as the 95th percentile and 95th percentile of a lognormal distribution were very similar, suggesting that the majority of predictions are lognormally distributed. Soil ingestion rate predictions varied from the lognormal distribution more than the predicted dust ingestion rates due to zero values (caused by sampled activity diaries that included no time spent outdoors). This finding is consistent with the findings of previous modeling efforts [16] and the authors believe this to be a natural result of random sampling of multiplicative factors from input distributions. Furthermore, there was no consistent age-dependent fit of predicted mean values of soil, total soil plus dust, and even dust ingestion rates by lognormal distributions across the ten different age groups we modeled.

**Fig. 2:**
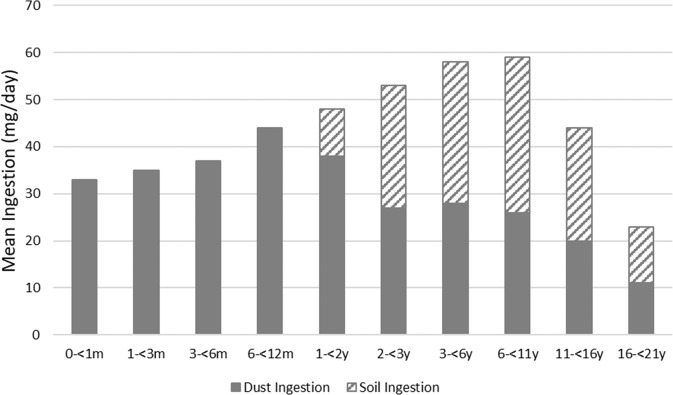
Annual means of total daily dust plus soil ingestion (mg/day) rates by age group. Exposure pathways influence potential exposures. Solid bars are contributions from dust ingestion; hatched bars are contributions from soil ingestion. More details in the text.

Figure 2 displays the means and contributions of the predicted soil and dust ingestion rates as a function of age. The data show that dust ingestion is the primary contributor to total soil and dust ingestion rates for children under one year old. However, soil and dust ingestion pathways were found to roughly contribute about equally to the predicted mean total annual soil plus dust ingestion rates for all ages of children above 2 years old.

**Fig. 3:**
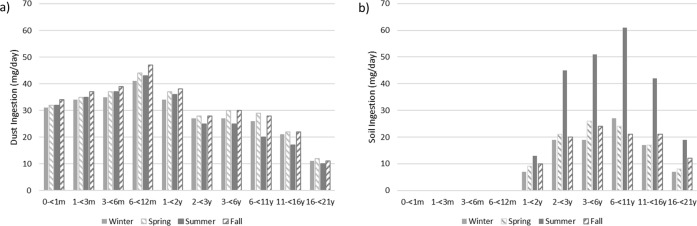
Comparison of mean (a) daily dust ingestion and (b) daily soil ingestion (mg/day) rates by age group and season. (Winter: December, January, and February; Spring: March, April, and May; Summer: June, July, and August; Fall: September, October, and November).

Figures 3a and b illustrate predicted dust and soil ingestion rates, respectively, as a function of season. We observed seasonal differences in soil and dust ingestion rates that were generally larger than the stochastic error. Basic summary statistics for each season are presented in Tables S7 through S10.

For toddlers and older age groups (2 to <21 years), the predicted average total soil plus dust ingestion rates are considerably higher in the summer months than during the winter months. This is influenced primarily by soil ingestion rates for age two and above due to the amount of time spent outdoors during the summer months based on time activity patterns in the diaries. More time spent outdoors typically leads to higher rates of soil ingestion by most children during normal play activities. Predicted dust ingestion rates have much less variability from season to season but are slightly reduced in the summer for ages two and above.

In conducting our sensitivity analysis, we chose four representative age groups (6 to <12 m, 2 to <3 y, 6 to <11 y, and 11 to <16 y). The results for the children 6–<12 m are presented in Table 3. Results for other age ranges are presented in Table S11. In summary, our analysis showed that for infants using pacifiers, four variables were most sensitive in influencing model predictions: carpet dust loading (dust_home_soft), pacifier drop frequency, probability of a blanket being used, and the floor-to-pacifier transfer fraction. For older children, four hand properties that collectively describe the amount of hand-to-mouth dust and soil transfer (i.e., the frequency of hand-to-mouth contact, the fraction of hand area mouthed, the amount of floor contacted by the hand, and the fraction of dust removed by each hand-to-mouth event) were the key drivers of predicted soil and dust ingestion rates, followed by soil-skin adherence (adherence_soil) and carpet dust loading (dust_home_soft).

**Table 3:** Sensitivity analyses results for modeled dust ingestion rates (mg/day) for infants 6–<12 months old.

Variable	Low exposure setting	High exposure setting	Ratio of high/low exposures	Sensitivity
Base Run: All set at Medians	17.42	17.42		
Dust_home_soft	3.52	97.53	27.71	**Decreasing variable sensitivity →**
Pacifier_drop	5.96	28.89	4.85	
P_blanket	8.01	26.82	3.35	
Pacifier_transfer	9.57	25.28	2.64	
P_pacifier	12.22	22.62	1.85	
Pacifier_frac_soft	12.37	22.43	1.81	
P_Home_soft	14.23	25.09	1.76	
Pacifier size	14.91	19.89	1.33	
F_blanket	15.53	19.31	1.24	

Note:

Most sensitive variables: High/Low exposure ratios are >2.

Marginally sensitive variables: High/Low exposure ratios are between 1.5 and 2.

Least sensitive variables: High/Low exposure ratios are between 1.1 and 1.5.

Not-sensitive variables: High/Low exposure ratios are <1.1 (not shown).

Results from the additional sensitivity analysis on the pacifier_washing, pacifier_transfer, and pacifier_drop variables are presented in Figure S1. Accounting for a pacifier being washed after an average of 10% or 30% of drops (represented by uniform distributions), linearly decreased ingestion by ~10% and 30%, respectively. This linear relationship between pacifier washing and reduction in dust ingestion for very young children highlights the impact of pacifier washing. Decreasing the pacifier_transfer variable that describes the transfer of surface dust to the pacifier when it is dropped to an average of 0.3 or 0.1, from the base scenario of 0.5 (all represented by uniform distributions), decreases the average ingestion 40% and 60%, respectively. This is acknowledged as a sensitive and uncertain variable. Finally, altering the average pacifier drop frequency from 8 to 7 or 6 drops per hour (maintaining the same normal distribution shape) decreases ingestion by 10% and 20%, respectively. The authors acknowledge this series of assumptions is uncertain and combined with previous sensitivity results suggest we could be potentially over- or underestimating infant’s exposures and warrants further research.

## Discussion

Soil and dust can each be a repository for a range of semi-volatile and particle bound pollutants, including flame retardants, pesticides, lead, and arsenic [4, 16, 18, 22, 23]. Intake of these chemicals via soil and dust ingestion can pose a health hazard, making quantifying the rate of ingestion, particularly for susceptible populations such as children, critical for conducting risk assessments. Published data on soil and dust ingestion rates for some child-specific age groups are limited or not available. Specifically, soil and dust ingestion rates for infants under 2 years and children greater than 7 years old are limited. To reduce this data gap, we modified the SHEDS-Soil/Dust model [13] to estimate soil and dust ingestion rates for individuals ranging in age from 0 to 21 years old by incorporating new algorithms representative of new exposure scenarios, available data, and extrapolating previously available data for various age groups. Additionally, we provide new data on seasonal effects on ingestion rates. Consequently, we can only compare a portion of our results to those previously published for certain age groups, based on either measurement or modeling-based methodologies. The following section summarizes our findings and compares our results to those available from past research publications.

### Comparison of findings to available results

**Fig. 4:**
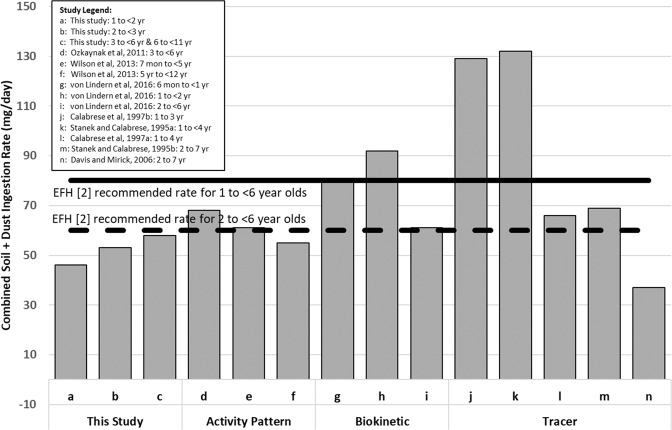
Comparison of mean soil plus dust ingestion rates as estimated in this study with published studies that employed the activity pattern, biokinetic, and tracer methodologies. U.S. EPA’s EFH [2] recommended soil and dust ingestion rates listed for comparison purposes. Note: von Lindern et al. [16] data are reported using the 50/25/15/10 partitioning approach. Tracer studies are reported using aluminum tracers. All values are reported as arithmetic means.

Previous studies on soil ingestion have utilized either the tracer, biokinetic modeling or activity pattern methodologies. These studies have mostly focused on estimating soil or soil plus dust ingestion rates for different groups of children within the age range 1–7 years old. Table 5-34 in the U.S. EPA’s EFH [2] summarizes soil and dust ingestion rates for children and adults from key studies using these different methodologies. Our analysis also included a tracer-based study by Davis and Mirick [9], not included in Table 5-34 [2], since it also included results for 12 children 2 to 7 years old. These study results are compared with our modeled annual mean total daily soil plus dust ingestion rates in Fig. 4.

The results from tracer studies show greater variation among studies. As mentioned earlier, the trace-element mass balance method suffers from various sources of uncertainty that could lead to considerable study-to-study variations. Estimated mean ingestion rates from four of the tracer soil and dust ingestion rate studies corresponding to the age groups between 1 and 4 years vary between 37 mg/day to 132 mg/day [4–6, 9]. For the broader age range of children 2 to 7 years old, a mean soil plus dust ingestion rate of 69 mg/day has been reported by Stanek and Calabrese [6], based on reanalysis of the Davis et al. [36] data. In contrast, our average annual mean SHEDS-Soil/Dust modeled soil and dust ingestion rates for the age groups including 1 to <3-year-old children is about 50 mg/day, increasing to 59 mg/day for 3 to <6-year-old children, and decreasing slightly for 6 to <11 year old children to 56 mg/day (see Table 2; Fig. 4). In general, our modeled estimates are lower than most of the results previously reported for children 1 to 7 years old. We should note, however, that seasonal differences might have also contributed to the observed overestimation of children’s true annual average soil ingestion rates in the tracer studies, since measurements were typically made either during the summer or early fall months, when soil ingestion rates are the highest.

In contrast, our results are consistent with the mean 52–67 mg/day values obtained from biokinetic and time activity pattern modeling studies for the 2–<6-year-old age group (von Lindern et al. [16] and Wilson et al. [14]). However, the biokinetic modeling results by von Lindern et al. [16], for the youngest group of children <1 year (86 mg/day, mean) and 1 year to 2 years (94 mg/day, mean) are considerably higher than annual mean modeling results of 44–48 mg/day for comparable age ranges. The von Lindern et al. [16] study was conducted using maximum seasonal blood lead concentrations and may be more directly comparable to the average summer ingestion rates for this study, 44 and 51 mg/day for the 6 to <12 month and 1 to <2 year age categories, respectively, which are still approximately two-folder lower. Again, differences in the methodology used, smaller sample size of the biokinetic study for young children and the specific geographic location of the von Lindern et al. [16] study (a former Pb Superfund site) may have played a role in differences found in the observed findings. Finally, our modeled estimates of annual average soil plus dust ingestion rates for 1 to <6 year old children (48 mg/day for 1 to <2 year, 52 mg/day for 2 to <3 year, and 59 mg/day for 3 to <6 year old children) are considerably lower than the 80 mg/day daily total soil plus dust ingestion value recommended for this age group of children in the U.S. EPA’s EFH [2].

### Modeling limitations

The SHEDS-Soil/Dust model applied during this research incorporates the observed inherent variability on most of the model inputs since SHEDS is a stochastic model that probabilistically implements model algorithms. However, some of the distributional inputs we used during our analysis are based on either limited data or are extrapolations made based on observations. In a few cases, we were unable to find any information for some variables of the enhanced SHEDS model and, as a result, had to make best professional judgment on the range of likely values for these variables. Clearly, such situations, due to assumed or known lack of knowledge, pose uncertainty in both the model inputs and the results. The impact of these uncertainties can be quantified to a certain degree by a comprehensive sensitivity and uncertainty analysis. Accordingly, we conducted a basic sensitivity analysis to identify those variables that are key drivers of the results. In addition to model input uncertainties, model results can be affected by scenario and model formulation or conceptual uncertainties. These are more difficult to quantify in the absence of other comparable or better models to be able to evaluate our results further. Our modeling of infants’ exposures only considered soil and dust exposures by pacifier use. We could not consider other plausible soil or dust exposure pathways in the absence of data to support modeling alternative soil and dust exposure pathways. Furthermore, for all age groups, the SHEDS-Soil/Dust model does not include soil or dust off-loading and re-loading pathways, except for particle removal by saliva when an objected is inserted into the mouth. For instance, the influence of rubbing hands or objects (such as blankets) on surfaces or clothes which may affect soil or dust loadings are not modeled by the current SHEDS-Soil/Dust model. Clearly, future research needs to focus on collecting additional data for reducing the input uncertainties as well as characterization of likely errors which may be attributed to scenario and model formulation uncertainties. Specific suggestions for future research to address these key modeling limitations are discussed below.

### Recommended research areas

Based on our modeling work, we recommend several future research activities to improve modeled estimates of soil and dust ingestion rates. These include:

1. Conducting new videography studies (indoors and outdoors) on infants under 1 year of age and for adolescents greater than 11 years old to determine key indoor and outdoor hand-to-mouth and object-to-mouth behaviors, including pacifier use and other uncharacterized behaviors by older children. Parental surveys around children’s pacifier use and mouthing behaviors as well as parental cleaning behaviors would be an alternative way to collect this data or strengthen videography data.
2. Collecting data on indoor dust loadings on blankets, baby clothing, household furniture, cribs, baby toys, and other various household surfaces where infants and toddlers may contact dust and relating these findings to dust loadings collected on floor and carpets simultaneously.
3. Characterizing mouthing behavior of older age groups of children and adolescents in multiple microenvironments, to account for touching or mouthing objects other than toys such as electronic appliances, sports equipment, materials used for hobbies.
4. Characterizing and quantifying other likely soil or dust exposure scenarios, such as handling and eating food items.
5. Conducting more targeted tracer studies that account for seasonal variations and enable evaluation of available modeling methods against new and more reliable measurement data.
6. Conducting simultaneous videography-based modeling studies along with better formulated tracer-based measurement studies to increase the confidence in both approaches. Currently, there are no U.S. studies that can be used to reliably compare and evaluate these distinct approaches.
7. Build from the work in Panagopoulos et al. [37] to explore how non-targeted analysis approaches can be used to identify novel chemicals useful as tracers.

## Supplementary information

Supplementary Material
