# Supplementary material for: Model based prediction of age-specific soil and dust ingestion rates for children
Source: J Expo Sci Environ Epidemiol. 2022 Jan 17;32(3):472–80. doi: 10.1038/s41370-021-00406-5 (PMC9119852; doi:10.1038/s41370-021-00406-5)
Supplement: Supplementary file 1 — Supplementary Material [file 41370_2021_406_MOESM1_ESM.docx]

# **Overview of the Enhanced SHEDS-Soil/Dust Model**

This research utilized a much broader application of an enhanced version of the previously reported mechanistic probabilistic SHEDS-Soil/Dust model described in Özkaynak et al. [[1](#_ENREF_1)]. Initial model modifications included minor updates to the code by incorporating any changes made within the Residential module [[2](#_ENREF_2)] of the latest SHEDS-Multimedia Multipathway Model (Version 4) code (<https://www.epa.gov/sites/production/files/2015-02/documents/shedsresidential_techmanual_2012.pdf>). Subsequent changes included algorithm and input modifications for predicting soil and dust ingestion rates corresponding to greater numbers of age categories of children. Specifically, the new model provides prediction of soil and dust ingestion rates for ten age categories between 0 and 21 years old (0 to <1m, 1 to <3m, 3 to <6m, 6m to <1y, 1 to <2y, 2 to <3y, 3 to <6y, 6 to <11y, 11 to <16y, 16 to <21 y), as defined in the U.S. EPA Guidance on selecting age categories for children for exposure assessment purposes [[3](#_ENREF_3)].

In the original SHEDS-Soil/Dust model [[1](#_ENREF_1)], data from all available videography studies of children engaged in everyday activities indoors and outdoors were used to quantify the contact frequencies with different surfaces, objects, and body parts [[4](#_ENREF_4), [5](#_ENREF_5)]. The basic algorithms of the enhanced SHEDS model also link diary information on sequential time spent in different locations and activities derived from the latest version of EPA’s Consolidated Human Activity Database (CHAD; <https://www.epa.gov/healthresearch/consolidated-human-activity-database-chad-use-human-exposure-and-health-studies-and>) with micro-activity data (e.g., hand-to-mouth frequency, hand-to-surface frequency information from videography studies) and microenvironmental surface/object soil or dust loadings, and other exposure factors (e.g., soil-to-skin adherence, saliva removal efficiency).

In the enhanced SHEDS-Soil/Dust model, apart from refining the age groupings, two other code modifications were implemented. The most important was developing and implementing an infant soil and dust ingestion modeling scenario within the new SHEDS-Soil/Dust model. Consideration was given to the fact that very young children may be placed on a blanket or similar surface, thus preventing their direct contact with the floor. Furthermore, infants and young children often use pacifiers. Although the SHEDS-Soil/Dust model previously contained an object-to-mouth exposure scenario, it did not explicitly include a pacifier exposure scenario. A new pacifier soil and dust ingestion algorithm was developed and implemented in the enhanced SHEDS-Soil/Dust model. As discussed in Özkaynak et al. [[1](#_ENREF_1)], the SHEDS model generates simulated individuals who collectively match the population age-sex distribution. Each simulated individual is assigned a set of activity diaries drawn from the CHAD human activity database [[6](#_ENREF_6)] and is assigned other relevant modeling parameters by randomly sampling from input distributions. Every individual is then followed through time, generally up to one year, and the model computes changes to their exposure at the diary event level. These correspond to the events as delineated by the CHAD diaries and range from one minute to one hour in duration. A year-long simulation will generally consist of 10,000 to 20,000 diary events. The SHEDS model output allows calculation of both within-person statistics (such as the average daily soil and dust ingestion rates) and across-person statistics (such as population percentiles).

# **Infants’ Behaviors by Pediatric Developmental Stages**

An online search yielded information on infant behaviors (e.g., hand-to-mouth behavior, thumb/finger sucking, teething) at different pediatric developmental stages to identify when these sorts of activities are dominant. We summarize key behaviors by various developmental stages after birth.

0-1 month: Thumb or finger sucking by infants begins within weeks of birth; sucking is an inborn or in utero reflex because it is how they eat [[7](#_ENREF_7), [8](#_ENREF_8)].

1-<3 months: Continued hand-to-mouth activities are observed during birth to 3 months [[9](#_ENREF_9)]. In addition to sucking fingers, newborns are also able to bring their hands or fists to their mouth and briefly suck the top of their fists [[10](#_ENREF_10), [11](#_ENREF_11)].

3-<6 months: Around 3 months old, infants may begin to lift objects, including bringing their thumb to their mouth [[12](#_ENREF_12)]. During this developmental phase, contact with surfaces and object/hand-to-mouth activities (steadily) increases [[9](#_ENREF_9)]. Infants in this age range can use their whole hands to grasp objects [[11](#_ENREF_11)] and can bring hands and objects into their mouth [[12](#_ENREF_12)]. At certain times, 3-4-month-old infants, while lying on their backs, may reach with both hands to their feet to play and even bring a toe briefly to their mouth [[13](#_ENREF_13)]. Most babies get their first teeth (lower central incisors) around 6 months, but some may get them as early as 3 months of age [[14](#_ENREF_14)].

This pediatric information suggests a steady increase in hand-to-mouth activities of children as their motor and coordination skills develop between the ages of birth to six months. Object-to-mouth behavior is often observed during 3-<6 months of age. Pacifier use is either observed or encouraged shortly after birth. Mouthing of baby toys, blankets or teething rings is often noted during 3-<6 months of age. However, babies may occasionally start this behavior prior to 3 months of age.

# **Sensitivity Analysis Methods**

Sensitivity analyses were performed for each age group and included the following steps:

- First, a base run was conducted with every input variable set to its median value and a median dust or soil+dust ingestion rate estimate was determined.
- Second, additional model runs were conducted, whereby a selected single input variable was changed to its 5^th^ percentile value, while leaving all other inputs at their median values.
- Third, the selected input variable was changed to its 95^th^ percentile value, again leaving every other input variable at its median value.
- The mean of the predicted ingestion rate (resulting from repeated SHEDS simulations across all available age-specific CHAD diaries) was then calculated for both the (low) 5^th^ percentile and (high) 95^th^ percentile model outputs.
- Finally, to quantify an index of the sensitivity of model results for each model variable evaluated, we calculated the ratio of high/low predicted means based on the variable-specific sensitivity results.

This process was repeated in the same manner for all inputs to the model. Sensitivity analysis model runs consisted of simulating 5,000 exposures of children for one year, except that the model runs performed with all variables at their median values had 10,000 children. For most variables, the low exposure run corresponds to the 5^th^ percentile, while the high exposure run to the 95^th^, but for variables marked with (*) these settings are reversed. These exceptions are made when input variables have a negative correlation with exposure, so a low variable setting produces a high exposure and vice versa.

An additional sensitivity analysis was conducted on three particularly uncertain variables to identify the impact of the choice of distribution. These variables are pacifier_washing, pacifier_transfer, and pacifier_drop.

In the absence of data, pacifier_washing was set to zero. To test the impact of this, two additional uniform distributions, 0 to 0.2 and 0 to 0.6, were evaluated. This suggests the pacifier was washed after either a maximum of 20% or 60% of drops.

In the base model runs, pacifier_transfer sampled from a uniform distribution of 0.25 to 0.75. In the sensitivity runs, narrower and less conservative uniform distributions of 0.1 to 0.5 and 0.05 to 0.25 were explored.

Finally, the frequency of pacifier drops (variable pacifier_drop) was tested with two alternate normal distributions: µ = 7, σ = 3 and µ = 6, σ = 3 compared to the baseline of µ = 8, σ = 3.

# **Tables of variables used in model simulations**

**Table S1. Distributions used for non-age specific model variables from** **Özkaynak et al. [**[**1**](#_ENREF_1)**]**

| **Variable** | **Description** | **Units** | **Distribution^1^** |
| --- | --- | --- | --- |
| Bath_days_max | Maximum # days between baths/showers | days | Multinomial (0.75, 0.14, 0.07, 0.01, 0.01, 0.01, 0.01)^2^ |
| Dust_home_hard | Dust loading on hard floors | μg/cm^2^ | Lognormal (42, 2.8) |
| Dust_home_soft | Dust loading on carpet | μg/cm^2^ | Lognormal (780, 2.9) |
| F_remove_bath | Fraction of loading removed by bath or shower | (-) | Uniform (0.9, 1.0) |
| F_remove_hand_mouth | Fraction of hand loading removed by one mouthing event | (-) | Beta (2, 8) |
| F_remove_hand_wash | Fraction of hand loading removed by hand washing | (-) | Uniform (0.3, 0.9) |
| F_remove_hour | Fraction of dermal loading removed by passage of time | (-) | Point (0)^3^ |
| F_transfer_dust_hands | Fraction of floor dust loading transferred to hands by contact | (-) | Triangle (0.01, 0.02, 0.03) |
| F_transfer_object_mouth | Fraction transferred from hands to mouth | (-) | Beta (2, 8) |
| Hand_contact_ratio | Ratio of floor area contacted hourly to the hand surface area | (1/hr) | Triangle (0, 2.4, 4.8) |
| Hand_load_max | Maximum combined soil and dust loading on hands | (μg/cm^2^) | Uniform (6000, 8000) |
| Hand_washes_per_day | Number of times per day the hands are washed | (1/day) | Lognormal (3.74, 2.63) |
| Object_floor_dust_ratio | Relative loadings of object and floor dust after contact | (-) | Uniform (0, 0.2) |
| P_home_hard | Probability of being in part of home with hard floor | (-) | Point (0.5) |
| P_home_soft | Probability of being in part of home with carpet | (-) | Point (0.5) |

^1^Parentheticals indicate the parameters for the statistical distribution used for the given variable. The parameters, by distribution, are: lognormal–GM, GSD; triangle–min, peak, max; uniform–min, max.

^2^Numbers in parentheticals are probabilities for the number of integer days between baths/showers ranging from 1 to 7 days.

^3^Conservative assumption

**Table S2. Distributions for the soil adherence variable**

| **Age Groups** | **Distribution** | **Type of Data Source** |
| --- | --- | --- |
| 0m-<6m | Point (0) {assume no soil contact} | Professional judgment |
| 1-<2y | Lognormal (0.055, 2.0)^1^ | Extrapolated from Özkaynak et al. [[1](#_ENREF_1)] |
| 2y-<21y | Lognormal (0.11, 2.0)^1^ | Published data for 3y old children [[1](#_ENREF_1)]; Professional judgment based on published data for ages other than 3y old |

^1^Parentheticals indicate the parameters for the statistical distribution used for the given variable. The parameters, by distribution, are: lognormal–GM, GSD.

**Table S3. Distributions for the hand_mouth_fraction variable**

| **Age Groups** | **Distribution^1^** |
| --- | --- |
| 0-<1m | Beta (1.7, 25) {μ = 0.064; extrapolated from Tsou et al. [[16](#_ENREF_16)]; assuming 50% of the mean of 0.12 for the 1y age group}^2^ |
| 1-<3m | Beta (2.5, 25) {μ = 0.09 ; extrapolated from Tsou et al. [[16](#_ENREF_16)]; assuming 75% of the mean of 0.12 for the 1y age group}^2^ |
| 3-<6m | Beta (2.5, 25) {μ = 0.09 ; extrapolated from Tsou et al. [[16](#_ENREF_16)]; assuming 75% of the mean of 0.12 for the 1y age group}^2^ |
| 6m-<1y | Beta (3.1, 25) (μ = 0.11 ; from Tsou et al. [[16](#_ENREF_16)]) |
| 1-<2y | Beta (3.6, 25) (μ = 0.12 ; from Tsou et al. [[16](#_ENREF_16)]) |
| 2-<3y | Beta (3.7, 25) (μ = 0.13 ; from Özkaynak et al. [[1](#_ENREF_1)] and Tsou et al. [[16](#_ENREF_16)]) |
| 3-<6y | Beta (2.4, 25) (μ = 0.09 ; extrapolated from Tsou et al. [[16](#_ENREF_16)]; note, this is 70% of the mean of 0.13 for the 2y age group) |
| 6-<11y | Beta (1.75, 25) {μ = 0.065 ; extrapolated from Tsou et al. [[16](#_ENREF_16)]; assuming 50% of the mean of 0.13 for the 2y age group}^2^ |
| 11-<16y | Beta (1.0, 25) {μ = 0.039 ; extrapolated from Tsou et al. [[16](#_ENREF_16)]; assuming 30% of the mean of 0.13 for the 2y age group}^2^ |
| 16-<21y | Beta (0.5, 25) {μ = 0.02 ; extrapolated from Tsou et al. [[16](#_ENREF_16)]; assuming 15% of the mean of 0.13 for the 2y age group}^2^ |

^1^Parentheticals indicate the parameters for the statistical distribution used for the given variable. Beta distributions used in this table are characterized by two shape parameters α, β of the specified Beta (α, β) distributions.

^2^The arithmetic means (μ) corresponding to the Beta distributions are provided inside the parentheses { } after each distribution. This information is used during the extrapolations made from relevant published data (as mentioned in the paper) by adjusting the arithmetic means of the original Beta distributions by keeping the β parameter of the original Beta distribution the same.

**Table S4. Distributions for the indoor and outdoor hand_mouth_frequency variables**

| **Age Groups** | **Indoor Distribution^1^** | **Outdoor Distribution^1^** |
| --- | --- | --- |
| 0-<1m | Weibull (1.28, 6) {μ = 5.6; extrapolated from Xue et al. [[4](#_ENREF_4)]; assuming 20% of the mean of 28 for the 3m age group}^2^ | Weibull (1.28, 2.4) (Estimated from Indoor distribution by assuming mean Outdoor/mean Indoor Hand-to-mouth frequency ratio is ~ 0.4; from Black et al. [[17](#_ENREF_17)])^2^ |
| 1-<3m | Weibull (1.28, 15) {μ = 14; extrapolated from Xue et al. [[4](#_ENREF_4)];assuming 50% of the mean of 28 for the 3m age group}^2^ | Weibull (1.28, 6) (Estimated from Indoor distribution by assuming mean Outdoor/mean Indoor Hand-to-mouth frequency ratio is ~ 0.4; from Black et al. [[17](#_ENREF_17)])^2^ |
| 3-<6m | Weibull (1.28, 30.19) {μ = 28; (Xue et al. [[4](#_ENREF_4)] | Weibull (1.28, 12) (Estimated from Indoor distribution by assuming mean Outdoor/mean Indoor Hand-to-mouth frequency ratio ~ 0.4 from Black et al. [[17](#_ENREF_17)])^2^ |
| 6m-<1y | Weibull (1.02, 19.01) (Xue et al. [[4](#_ENREF_4)]) | Weibull (1.39, 15.98) (Xue et al. [[4](#_ENREF_4)]) |
| 1-<2y | Weibull (0.91, 18.79) (Xue et al. [[4](#_ENREF_4)]) | Weibull (0.98, 13.76) (Xue et al. [[4](#_ENREF_4)]) |
| 2-<3y | Weibull (0.76, 11.04) (Xue et al. [[4](#_ENREF_4)]) | Weibull (0.56, 3.41) (Xue et al. [[4](#_ENREF_4)]) |
| 3-<6y | Weibull (0.75, 12.59) (Xue et al. [[4](#_ENREF_4)]) | Weibull (0.55, 5.53) (Xue et al. [[4](#_ENREF_4)]) |
| 6-<11y | Weibull (1.36, 7.34) {μ = 6.7 ;(Xue et al. [[4](#_ENREF_4)] | Weibull (0.49, 1.47) {μ = 3 ; (Xue et al. [[4](#_ENREF_4)]} |
| 11-<16y | Weibull (1.36, 5.5) {μ = 5 and σ = 3.7; extrapolated from Xue et al. [[4](#_ENREF_4)]; assuming 75% of the mean of 6.7 for the 6y age group}^2^ | Weibull (0.49, 1.1) {μ = 2.3; extrapolated from Xue et al. [[4](#_ENREF_4)]; assuming 75% of the mean of 3 for the 6y age group}^2^ |
| 16-<21y | Weibull (1.36, 3.7) {μ = 3.4; extrapolated from Xue et al. [[4](#_ENREF_4)]; assuming 50% of the mean of 6.7 for the 6y age group}^2^ | Weibull (0.49, 0.74) {μ = 1.5 ; extrapolated from Xue et al. [[4](#_ENREF_4)]; assuming 50% of the mean of 3 for the 6y age group}^2^ |

^1^Parentheticals indicate the parameters for the statistical distribution used for the given variable. The parameters of the Weibull distribution are shape and scale.

^2^Bracketed μ are the arithmetic means of the Weibull distributions specified and were used to extrapolate to age for which data was not identified.

**Table S5. Distributions for the object_mouth_area variable**

| **Age Groups** | **Distribution^1,2^** |
| --- | --- |
| 0-<1m | Exponential (1, 3.3) (Assume maximum = 16.5 cm^2^) |
| 1-<3m | Exponential (1, 3.3) (Assume maximum = 16.5 cm^2^) |
| 3-<6m | Exponential (1, 4.4) (Assume maximum = 22 cm^2^) |
| 6m-<1y | Exponential (1, 4.4) (Assume maximum = 22 cm^2^) |
| 1-<2y | Exponential (1, 6.7) (Assume maximum = 33.5 cm^2^) |
| 2-<3y | Exponential (1, 6.7) (Assume maximum = 33.5 cm ^2^) |
| 3-<6y | Exponential (1, 10) (Assume maximum = 50 cm^2^) [[1](#_ENREF_1)] |
| 6-<11y | Exponential (1, 10) (Assume maximum = 50 cm^2^) |
| 11-<16y | Exponential (1, 10) (Assume maximum = 50 cm^2^) |
| 16-<21y | Exponential (1, 10) (Assume maximum = 50 cm^2^) |

^1^Parentheticals indicate the parameters for the statistical distribution used for the given variable. The parameters for the exponential distribution are min, mean, max.

^2^Distribution for children aged 3-<6y is from Özkaynak et al. [[1](#_ENREF_1)]. Distributions for all other age ranges based on professional judgment and extrapolation from this previously published data.

**Table S6. Distributions for the indoor and outdoor object_mouth_frequency variables**

| **Age Groups** | **Indoor Distribution** | **Outdoor Distribution** |
| --- | --- | --- |
| 0-<1m | Point (0) {Note: for newborns only the pacifier object mouthing is considered}^1^ | Point (0) {assume pacifier contact only, for newborns while outdoor}^1^ |
| 1-<3m | Weibull (0.74, 2.81) {μ = 3.4 extrapolated from Xue et al. [[5](#_ENREF_5)]; assuming 30% of the mean of 11.4 for the 3m age group}^1,2^ | Weibull (0.93,1.97) (μ=1.97; extrapolated by assuming 30% of the mean value for the 3m age category; (Xue et al. [[5](#_ENREF_5)])^2^; |
| 3-<6m | Weibull (0.74, 9.44) {μ=11.4; μ=from Xue et al. [5] with the pacifier adjustment factor of 0.96 applied to the scale parameter}^2^ | Weibull (0.93,6.6) (μ=6.8; Estimated from the Indoor distribution by assuming mean Outdoor/mean Indoor hand-to-mouth ratio of ~ 0.6, based on data from Xue et al. [[5](#_ENREF_5)] for the 1-2y age category)^2^ |
| 6m-<1y | Weibull (1.66, 21.81) {assumed to be same as for the 1y age category in Xue et al. [[5](#_ENREF_5)] with the pacifier adjustment factor of 0.96 applied to the scale parameter} | Weibull (0.93, 8.24) {assumed to be same as for the 1y age category in Xue et al. [[5](#_ENREF_5)] with the pacifier adjustment factor of 0.96 applied to the scale parameter} |
| 1-<2y | Weibull (1.39, 14.92) (from Xue et al. [[5](#_ENREF_5)] with the pacifier adjustment factor of 0.96 applied to the scale parameter) | Weibull (0.93, 8.24) (from Xue et al. [[5](#_ENREF_5)] with the pacifier adjustment factor of 0.96 applied to the scale parameter) |
| 2-<3y | Weibull (1.36, 10.75) (Xue et al. [[5](#_ENREF_5)]) | Weibull (0.64, 6.15) (Xue et al. [[5](#_ENREF_5)]) |
| 3-<6y | Weibull (0.58, 6.90) (Xue et al. [[5](#_ENREF_5)]) | Weibull (0.55, 5.38) (Xue et al. [[5](#_ENREF_5)]) |
| 6-<11y | Weibull (0.85, 1.04) (Xue et al. [[5](#_ENREF_5)]) | Weibull (0.55, 1.10) (Xue et al. [[5](#_ENREF_5)]) |
| 11-<16y | Weibull (0.85, 0.78) { extrapolated from Xue et al. [[5](#_ENREF_5)]; assuming 75% of the mean of 1.1 for the 6y age group}^2^ | Weibull (0.55, 0.83) { extrapolated from Xue et al. [[5](#_ENREF_5)]; assuming 75% of the mean of 1.9 for the 6y age group}^2^ |
| 16-<21y | Weibull (0.85, 0.52) { extrapolated from Xue et al. [[5](#_ENREF_5)]; assuming 50% of the mean of 1.1 for the 6y age group}^2^ | Weibull (0.55, 0.55) { extrapolated from Xue et al. [[5](#_ENREF_5)]; assuming 50% of the mean of 1.9 for the 6y age group}^2^ |

^1^ Source: Professional judgment

^2^ Source: Extrapolated from published data while assuming the same Weibull shape parameter

# **Additional Soil and Dust Ingestion Rate Results**

**Table S7. Seasonal mean total daily dust and soil ingestion (mg/day) predictions for spring**

| **Age Group** | **Dust Plus Soil Ingestion** | | | | | | **Dust Ingestion** | | | | | | **Soil Ingestion** | | | | | |
| --- | --- | --- | --- | --- | --- | --- | --- | --- | --- | --- | --- | --- | --- | --- | --- | --- | --- | --- |
|  | **Mean** | **Std. Dev** | **GM** | **GSD** | **Median** | **95th %ile** | **Mean** | **Std. Dev** | **GM** | **GSD** | **Median** | **95th %ile** | **Mean** | **Std. Dev** | **GM** | **GSD** | **Median** | **95th %ile** |
| 0-<1m | 32 | 46 | 18 | 3 | 18 | 103 | 32 | 46 | 18 | 3 | 18 | 103 | 0 | 0 | . | . | 0 | 0 |
| 1-<3m | 36 | 56 | 20 | 2.9 | 19.3 | 120 | 36 | 56 | 20 | 2.9 | 19.3 | 120 | 0 | 0 | . | . | 0 | 0 |
| 3-<6m | 37 | 51 | 21 | 2.8 | 21.5 | 119 | 37 | 51 | 21 | 2.8 | 21.5 | 119 | 0 | 0 | . | . | 0 | 0 |
| 6m-<1y | 44 | 75 | 24 | 3 | 24.6 | 150 | 44 | 75 | 24 | 3 | 24.6 | 150 | 0 | 0 | . | . | 0 | 0 |
| 1-<2y | 48 | 65 | 31 | 2.5 | 30.1 | 143 | 38 | 61 | 23 | 2.7 | 22.4 | 123 | 9 | 19 | 3.8 | 6 | 3.1 | 40 |
| 2-<3y | 50 | 74 | 26 | 3.3 | 27.5 | 165 | 28 | 53 | 14 | 3.3 | 14.5 | 94 | 22 | 45 | 7.2 | 7.5 | 7 | 95 |
| 3-<6y | 57 | 75 | 31 | 3.3 | 32.5 | 190 | 30 | 46 | 15 | 3.5 | 16.2 | 105 | 27 | 52 | 8.8 | 6.9 | 8.9 | 115 |
| 6-<11y | 50 | 72 | 25 | 3.4 | 27.1 | 170 | 28 | 46 | 14 | 3.5 | 14.6 | 101 | 22 | 47 | 6.3 | 7.7 | 6.9 | 86 |
| 11-<16y | 40 | 70 | 16 | 4.1 | 17.7 | 151 | 24 | 49 | 9.3 | 4.2 | 10 | 88 | 16 | 39 | 3.4 | 9.9 | 3.6 | 70 |
| 16-<21y | 21 | 53 | 6 | 5.3 | 6.2 | 86 | 12 | 33 | 3.6 | 5.5 | 3.9 | 50 | 8 | 36 | 1.2 | 9.7 | 1.2 | 36 |

**Table S8. Seasonal mean total daily dust and soil ingestion (mg/day) predictions for summer**

| **Age Group** | **Dust Plus Soil Ingestion** | | | | | | **Dust Ingestion** | | | | | | **Soil Ingestion** | | | | | |
| --- | --- | --- | --- | --- | --- | --- | --- | --- | --- | --- | --- | --- | --- | --- | --- | --- | --- | --- |
|  | **Mean** | **Std. Dev** | **GM** | **GSD** | **Median** | **95th %ile** | **Mean** | **Std. Dev** | **GM** | **GSD** | **Median** | **95th %ile** | **Mean** | **Std. Dev** | **GM** | **GSD** | **Median** | **95th %ile** |
| 0-<1m | 32 | 44 | 18 | 2.9 | 18.5 | 104 | 32 | 44 | 18 | 2.9 | 18.5 | 104 | 0 | 0 | . | . | 0 | 0 |
| 1-<3m | 35 | 55 | 20 | 2.9 | 19.6 | 117 | 35 | 55 | 20 | 2.9 | 19.6 | 117 | 0 | 0 | . | . | 0 | 0 |
| 3-<6m | 36 | 48 | 22 | 2.8 | 21.8 | 113 | 36 | 48 | 22 | 2.8 | 21.8 | 113 | 0 | 0 | . | . | 0 | 0 |
| 6m-<1y | 44 | 69 | 25 | 2.9 | 25.4 | 137 | 44 | 69 | 25 | 2.9 | 25.4 | 137 | 0 | 0 | . | . | 0 | 0 |
| 1-<2y | 51 | 61 | 33 | 2.5 | 32.5 | 155 | 36 | 51 | 21 | 2.7 | 20.9 | 116 | 15 | 33 | 4.8 | 6.6 | 5.1 | 62 |
| 2-<3y | 70 | 88 | 39 | 3.1 | 41.5 | 224 | 23 | 39 | 12 | 3.2 | 12.5 | 79 | 47 | 73 | 17 | 6.4 | 21 | 178 |
| 3-<6y | 81 | 112 | 42 | 3.3 | 43.9 | 280 | 25 | 40 | 12 | 3.5 | 12.7 | 85 | 57 | 100 | 19 | 6.8 | 21.5 | 232 |
| 6-<11y | 76 | 124 | 36 | 3.6 | 37.7 | 265 | 19 | 33 | 8.3 | 3.8 | 8.8 | 68 | 57 | 115 | 20 | 5.3 | 22.3 | 220 |
| 11-<16y | 57 | 109 | 22 | 4.3 | 23.3 | 210 | 17 | 41 | 6.1 | 4.5 | 6.5 | 65 | 40 | 91 | 10 | 6.6 | 11.9 | 160 |
| 16-<21y | 30 | 69 | 8.1 | 5.5 | 8.3 | 127 | 10 | 23 | 2.9 | 5.6 | 3.1 | 42 | 20 | 59 | 2.9 | 9.3 | 3.2 | 90 |

**Table S9. Seasonal mean total daily dust and soil ingestion (mg/day) predictions for fall**

| **Age Group** | **Dust Plus Soil Ingestion** | | | | | | **Dust Ingestion** | | | | | | **Soil Ingestion** | | | | | |
| --- | --- | --- | --- | --- | --- | --- | --- | --- | --- | --- | --- | --- | --- | --- | --- | --- | --- | --- |
|  | **Mean** | **Std. Dev** | **GM** | **GSD** | **Median** | **95th %ile** | **Mean** | **Std. Dev** | **GM** | **GSD** | **Median** | **95th %ile** | **Mean** | **Std. Dev** | **GM** | **GSD** | **Median** | **95th %ile** |
| 0-<1m | 34 | 47 | 19 | 3 | 19.2 | 109 | 34 | 47 | 19 | 3 | 19.2 | 109 | 0 | 0 | . | . | 0 | 0 |
| 1-<3m | 37 | 58 | 21 | 2.9 | 20.9 | 124 | 37 | 58 | 21 | 2.9 | 20.9 | 124 | 0 | 0 | . | . | 0 | 0 |
| 3-<6m | 39 | 53 | 23 | 2.8 | 22.6 | 124 | 39 | 53 | 23 | 2.8 | 22.6 | 124 | 0 | 0 | . | . | 0 | 0 |
| 6m-<1y | 46 | 76 | 26 | 3 | 26.2 | 149 | 46 | 76 | 26 | 3 | 26.2 | 149 | 0 | 0 | . | . | 0 | 0 |
| 1-<2y | 48 | 63 | 31 | 2.5 | 31.6 | 144 | 39 | 58 | 23 | 2.7 | 23.1 | 119 | 10 | 23 | 2.8 | 10.2 | 3 | 40 |
| 2-<3y | 46 | 70 | 24 | 3.3 | 25.9 | 153 | 27 | 49 | 14 | 3.3 | 14.2 | 88 | 20 | 46 | 4.1 | 12 | 5 | 84 |
| 3-<6y | 53 | 70 | 29 | 3.3 | 30.3 | 176 | 30 | 45 | 15 | 3.4 | 16.2 | 103 | 23 | 46 | 5.6 | 11.3 | 7.4 | 102 |
| 6-<11y | 48 | 70 | 24 | 3.5 | 26 | 166 | 28 | 48 | 13 | 3.5 | 14 | 94 | 21 | 44 | 4.6 | 10.6 | 5.9 | 89 |
| 11-<16y | 43 | 74 | 18 | 4.1 | 19.2 | 160 | 23 | 47 | 9.1 | 4.2 | 9.9 | 86 | 20 | 46 | 4.1 | 9.4 | 5.1 | 90 |
| 16-<21y | 24 | 59 | 7 | 5.4 | 7.3 | 97 | 12 | 32 | 3.4 | 5.5 | 3.7 | 47 | 13 | 42 | 1.7 | 10.7 | 1.8 | 54 |

Table S10. Seasonal mean total daily dust and soil ingestion (mg/day) predictions for winter

| **Age Group** | **Dust Plus Soil Ingestion** | | | | | | **Dust Ingestion** | | | | | | **Soil Ingestion** | | | | | |
| --- | --- | --- | --- | --- | --- | --- | --- | --- | --- | --- | --- | --- | --- | --- | --- | --- | --- | --- |
|  | **Mean** | **Std. Dev** | **GM** | **GSD** | **Median** | **95th %ile** | **Mean** | **Std. Dev** | **GM** | **GSD** | **Median** | **95th %ile** | **Mean** | **Std. Dev** | **GM** | **GSD** | **Median** | **95th %ile** |
| 0-<1m | 31 | 46 | 17 | 3.1 | 17 | 104 | 31 | 46 | 17 | 3.1 | 17 | 104 | 0 | 0 | . | . | 0 | 0 |
| 1-<3m | 34 | 54 | 18 | 3 | 18.7 | 115 | 34 | 54 | 18 | 3 | 18.7 | 115 | 0 | 0 | . | . | 0 | 0 |
| 3-<6m | 35 | 48 | 20 | 2.9 | 20.3 | 112 | 35 | 48 | 20 | 2.9 | 20.3 | 112 | 0 | 0 | . | . | 0 | 0 |
| 6m-<1y | 42 | 72 | 23 | 3.1 | 22.9 | 131 | 42 | 72 | 23 | 3.1 | 22.9 | 131 | 0 | 0 | . | . | 0 | 0 |
| 1-<2y | 43 | 57 | 27 | 2.6 | 26.7 | 133 | 36 | 54 | 21 | 2.8 | 20.8 | 116 | 7 | 16 | 2.2 | 8.5 | 1.6 | 31 |
| 2-<3y | 44 | 69 | 23 | 3.3 | 23.7 | 152 | 25 | 49 | 13 | 3.3 | 13.2 | 86 | 19 | 46 | 4 | 13.1 | 3.8 | 88 |
| 3-<6y | 46 | 69 | 24 | 3.3 | 25.2 | 144 | 27 | 42 | 14 | 3.5 | 14.6 | 96 | 18 | 48 | 4.7 | 10.8 | 5 | 74 |
| 6-<11y | 51 | 76 | 175 | 26.4 | 24.9 | 3.5 | 25 | 41 | 12 | 3.6 | 12.4 | 90 | 25 | 57 | 7.3 | 8.2 | 8.2 | 105 |
| 11-<16y | 38 | 66 | 16 | 4.1 | 17.3 | 143 | 22 | 44 | 8.5 | 4.2 | 9.3 | 79 | 16 | 39 | 3.9 | 8 | 4.3 | 70 |
| 16-<21y | 18 | 46 | 5 | 5.5 | 5.2 | 73 | 12 | 28 | 3.3 | 5.5 | 3.6 | 46 | 7 | 30 | 0.7 | 12.5 | 0.5 | 28 |

**Table S11. Results from sensitivity analyses for modeled soil and dust ingestion rates (mg/day) for children aged 2-<3 years, 6-<11 years, and 11-<16 years**

|  | **2-<3 Years Old** | | | **6-<11 Years Old** | | | **11-<16 Years Old** | | |
| --- | --- | --- | --- | --- | --- | --- | --- | --- | --- |
| **Variable** | **Low Exposure** | **High Exposure** | **Ratio of High/Low Exposures** | **Low Exposure** | **High Exposure** | **Ratio of High/Low Exposures** | **Low Exposure** | **High Exposure** | **Ratio of High/Low Exposures** |
| Base Run: All set to median values | 31.3 | 31.3 |  | 29.3 | 29.3 |  | 18.4 | 18.4 |  |
| Hand_mouth_fraction | 14.6 | 51.8 | 3.56 | 6.3 | 72.1 | 11.5 | 1.88 | 67.5 | 35.9 |
| Hand_mouth_freq | 4.27 | 66.0 | 15.5 | 5.6 | 71.7 | 12.7 | 3.47 | 49.7 | 14.3 |
| F_remove_hand_mouth | 11.2 | 50.8 | 4.52 | 8.2 | 54.7 | 6.71 | 4.95 | 38.0 | 7.68 |
| Hand_contact_ratio | 11.9 | 50.5 | 4.26 | 9.7 | 48.6 | 5.02 | 6.19 | 30.5 | 4.92 |
| Dust_home_soft | 20.7 | 92.1 | 4.45 | 20.1 | 80.5 | 4.01 | 12.3 | 53.7 | 4.36 |
| Adherence_soil | 19.2 | 68.9 | 3.59 | 17.1 | 65.1 | 3.82 | 11.2 | 40.6 | 3.63 |
| Hand_washes_per_day (*) | 22.7 | 36.9 | 1.62 | 20.7 | 34.3 | 1.66 | 12.9 | 22.4 | 1.74 |
| P_home_soft | 26.2 | 36.6 | 1.40 | 26.2 | 31.6 | 1.20 | 16.6 | 20.3 | 1.23 |
| Object_mouth_area | 29.2 | 39.1 | 1.34 |  |  |  | 17.9 | 19.8 | 1.11 |
| F_transfer_dust_hands | 27.5 | 35.2 | 1.28 | 25.3 | 33.0 | 1.30 | 15.8 | 20.9 | 1.32 |
| Object_mouth_freq | 28.8 | 34.6 | 1.20 | 28.5 | 32.4 | 1.13 | 17.9 | 21.2 | 1.18 |
| F_remove_hand_wash (*) | 29.2 | 34.1 | 1.17 | 26.1 | 31.8 | 1.18 | 17.1 | 20.4 | 1.20 |
| F_transfer_object_mouth | 29.0 | 33.7 | 1.16 |  |  |  |  |  |  |
| Dust_hard |  |  |  | 28.5 | 31.8 | 1.11 | 18.0 | 20.2 | 1.13 |

Variables shaded in light gray had a ratio of high/low >2, medium gray 1.5 – 2, and dark gray <1.5. Variables with a ratio < 1.1 are not shown.

Variables with a ratio < 1.1 are not shown. Variables not shown are: Bath_days_max, F_remove_bath, F_remove_hour, Hand_load_max, Object_floor_dust_ratio, and P_home_hard.


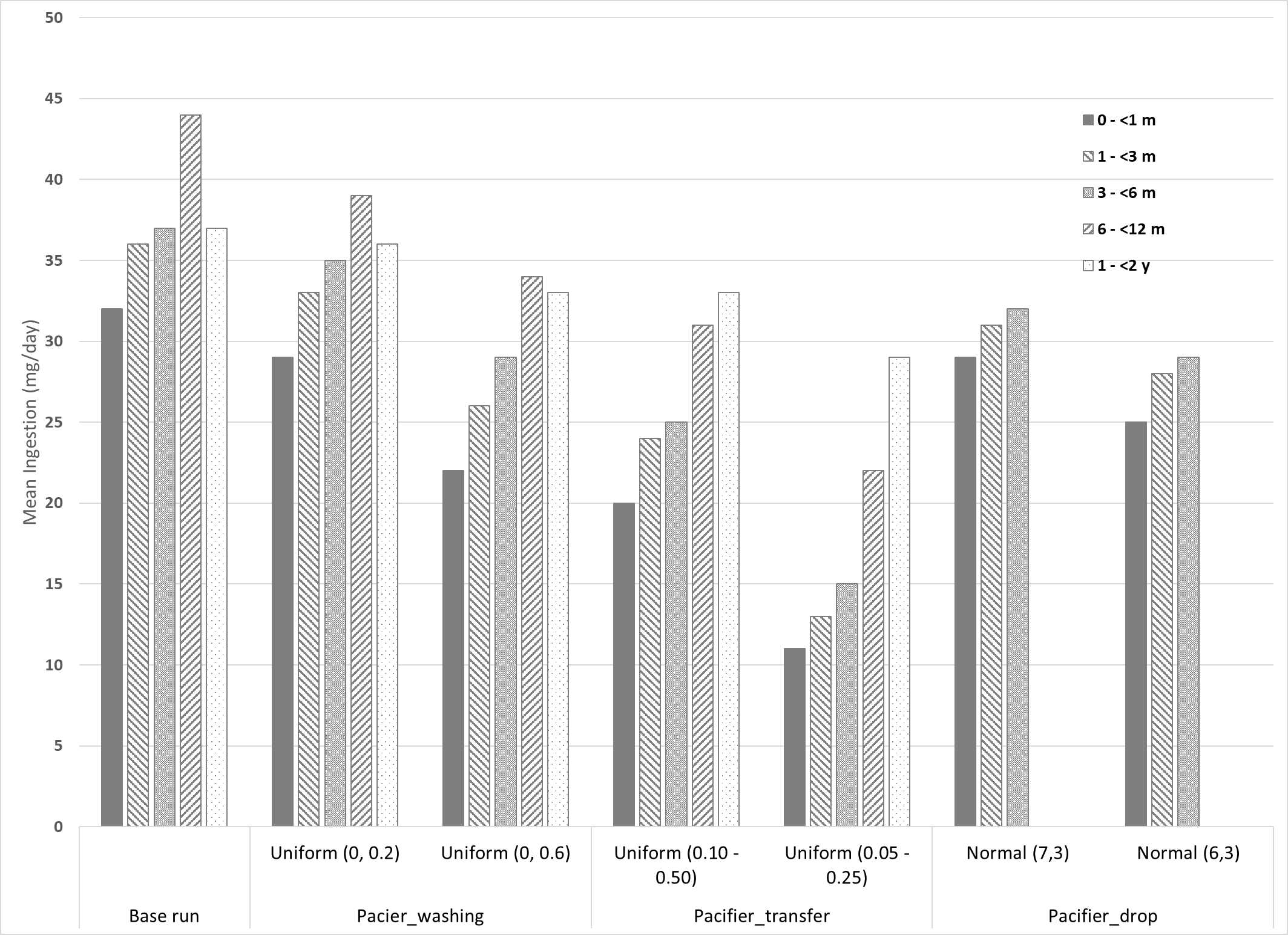


**Figure S1. Results from sensitivity analyses for modeled soil and dust ingestion rates (mg/day) for the pacitifer_washing, pacifier_transfer, and pacifier_drop scenarios.**

# **References**

1. Özkaynak H, Xue JP, Zartarian VG, Glen G, Smith L. Modeled estimates of soil and dust ingestion rates for children. Risk Anal. 2011;31(4):592-608.

2. Glen G, Zartarian V, Smith L, Xue J. The Stochastic Human Exposure and Dose Simulation Model for multimedia, multipathway chemicals (SHEDS-multimedia): Residential module. SHEDS-Residential version 4. Technical manual. 2012. <https://www.epa.gov/sites/production/files/2015-02/documents/shedsresidential_techmanual_2012.pdf>.

3. U.S. Environmental Protection Agency (USEPA). Exposure Factors Handbook. Washington, DC: U.S. Environmental Protection Agency; 2005. <https://www.epa.gov/expobox/about-exposure-factors-handbook>.

4. Xue J, Zartarian V, Moya J, Freeman N, Beamer P, Black K, et al. A meta-analysis of children's hand-to-mouth frequency data for estimating nondietary ingestion exposure. Risk Anal. 2007;27(2):411-20.

5. Xue J, Zartarian V, Tulve N, Moya J, Freeman N, Auyeung W, et al. A meta-analysis of children's object-to-mouth frequency data for estimating non-dietary ingestion exposure. J Expo Sci Environ Epidemiol. 2010;20(6):536-45.

6. McCurdy T, Glen G, Smith L, Lakkadi Y. The National Exposure Research Laboratory's consolidated Human Activity Database. J Expo Sci Environ Epidemiol. 2000;10(6):566-78.

7. What to Expect. Thumb-sucking baby: Is it okay for newborns to suck their thumbs? ; 2016. <https://www.whattoexpect.com/first-year/ask-heidi/thumb-sucking-baby.aspx> Accessed 20 Dec 2016.

8. Rochat P, Bass EM, L.B. H. Oropharyngeal control of hand-to-mouth coordination in newborn infants. Dev Psychol. 1988;24(4):459–63.

9. Moya J, Bearer CF, Etzel RA. Children's behavior and physiology and how it affects exposure to environmental contaminants. Pediatrics. 2004;113(4 Suppl):996-1006.

10. Pathways. 0-3 months. 2016. <https://pathways.org/growth-development/0-3-months/> Accessed 20 Dec 2016.

11. U.S. Department of Agriculture (USDA). Chapter 2: Development of infant feeding skills. United States Department of Agriculture, Food and Nutrition Services; 2009. <https://wicworks.fns.usda.gov/wicworks/Topics/FG/Chapter2_DevelopmentofFeedingSkills.pdf> Accessed 20 Dec 2016.

12. Parents. How baby's hand skills develop. 2016. <http://www.parents.com/baby/development/physical/how-babys-hand-skills-develop/> Accessed 20 Dec 2016.

13. Pathways. 4-6 months. 2016. <https://pathways.org/growth-development/4-6-months/> Accessed 20 Dec 2016.

14. Parents. Baby teething symptoms: Is your infant cutting teeth? ; 2016. <http://www.parenting.com/article/guide-teething-symptoms> Accessed 20 Dec 2016.

15. Sippola MR, Sextro RG, Thatcher TL. Measurements and modeling of deposited particle transport by foot traffic indoors. Environ Sci Technol. 2014;48(7):3800-7.

16. Tsou MC, Özkaynak H, Beamer P, Dang W, Hsi HC, Jiang CB, et al. Mouthing activity data for children age 3 to <6 years old and fraction of hand area mouthed for children age <6 years old in Taiwan. J Expo Sci Environ Epidemiol. 2018;28(2):182-92.

17. Black K, Shalat SL, Freeman NCG, Jimenez M, Donnelly KC, Calvin JA. Children's mouthing and food-handling behavior in an agricultural community on the US/Mexico border. J Exp Anal Environ Epidemiol. 2005;15(3):244-51.
